# Differentiating obesity with and without prediabetes through skin findings: results from PREVIEW sub-study

**DOI:** 10.3389/fendo.2026.1796607

**Published:** 2026-04-01

**Authors:** Razvigor Darlenski, Vesselina Mihaylova, Karen Manuelyan, Denitza Zheleva, Georgi Bogdanov, Ivan Bogdanov, Todor Kundurzhiev, Pavlina Gateva, Mikael Fogelholm, Anne Raben, Teodora Handjieva-Darlenska

**Affiliations:** 1 Department of Dermatology and Venereology, Acibadem Cityclinic Tokuda Hospital, Sofia, Bulgaria; 2 Department of Dermatology and Venereology, Trakia University, Stara Zagora, Bulgaria; 3 Department of Pharmacology and Toxicology, Medical Faculty, Medical University, Sofia, Bulgaria; 4 Faculty of Public Health, Medical University, Sofia, Bulgaria; 5 Department of Food and Environmental Sciences, University of Helsinki, Helsinki, Finland; 6 Department of Nutrition, Exercise and Sports, SCIENCE, University of Copenhagen, Copenhagen, Denmark

**Keywords:** diabetes, insulin resistance, overweight, plantar hyperkeratosis, skin tag

## Abstract

**Objective:**

To explore whether specific cutaneous findings are associated with prediabetes among adults with overweight or obesity, and may assist in clinical risk stratification.

**Materials and methods:**

This single-center, cross-sectional analysis included 191 adults with overweight or obesity (BMI ≥25 kg/m²) recruited during screening visits of the Bulgarian PREVIEW cohort; 151 participants had prediabetes, and 40 did not. A trained dermatologist performed a standardized skin examination. Xerosis, pruritus, and yellowish skin tone were assessed using 10-point visual scales. The prevalence of dermatological conditions was compared between groups, and exploratory binary logistic regression was used to examine associations with prediabetes.

**Results:**

Horseshoe-like plantar hyperkeratosis (HSLPH) was more frequent in participants with prediabetes than in those without (25.2% vs. 10.0%, p = 0.039). The presence of HSLPH was associated with higher odds of prediabetes (odds ratio 3.03, 95% confidence interval 1.01–9.06). HSLPH showed high specificity (90%) but low sensitivity (25%). The prevalence of other common skin conditions, including skin tags, onychomycosis, signs of chronic venous insufficiency, seborrheic dermatitis, and tinea pedis, did not differ between groups. Skin tag counts were not associated with body weight or BMI. No significant differences were observed in xerosis, pruritus, or yellowish skin tone scores.

**Conclusion:**

In adults with overweight or obesity, HSLPH was the only cutaneous finding associated with prediabetes and behaved as a specific but insensitive clinical sign. Its presence may serve as a visible clinical red flag prompting metabolic evaluation, whereas its absence does not exclude dysglycemia. Other common obesity-related skin findings were not discriminatory for prediabetes in this cohort.

## Introduction

Diabetes is a systemic disease that affects multiple organs and systems, including the skin (1, 2). The most frequently encountered cutaneous manifestations associated with diabetes and impaired glucose metabolism include diabetic dermopathy, acanthosis nigricans, necrobiosis lipoidica, rubeosis faciei, and insulin-resistance–related skin thickening, particularly acanthosis nigricans, together with increased susceptibility to bacterial and fungal infections (1). Rarer entities such as diabetic bullae or scleredema adultorum are typically observed in longstanding or poorly controlled disease and are not representative of early dysglycemia (2).

Overweight and obesity are significant risk factors for diabetes and commonly precede the development of diabetes mellitus (3, 4). Obesity induces insulin resistance and beta-cell dysfunction, which are central to the pathogenesis of type 2 diabetes (5, 6). More than 80% of individuals with prediabetes in the US are overweight or lining with obesity, providing evidence on the epidemiological level of the association between excess adiposity and dysglycemia (7).

The skin of patients living with obesity shows specific changes, including skin tags and horseshoe-like plantar hyperkeratosis (8). A link between skin tags, insulin resistance, and leptin levels has been established (9). The exact mechanism underlying the development of dermatological signs of impaired glucose tolerance and insulin resistance remains to be elucidated.

Prediabetes represents an intermediate disturbance in glucose regulation, defined by fasting plasma glucose of 100–125 mg/dL, 2-hour plasma glucose of 140–199 mg/dL after an oral glucose tolerance test, or HbA1c between 5.7–6.4% according to ADA criteria (3). Meeting multiple criteria increases the likelihood of progression to diabetes, with annual conversion rates ranging from about 6% to 18% and cumulative incidence exceeding 30% over a decade (7, 10). The mean duration of the prediabetic state is 6–10 years, but progression can be faster when impaired glucose tolerance is present. Within five years, one-fifth of individuals with HbA1c-defined prediabetes develop diabetes (10, 11).

Recognizing such “markers” that are easy to objectify could be used in clinical practice, especially when laboratory investigation is not applicable. The literature search yielded no data on the specific skin findings in prediabetes. In the current observational study, we hypothesized that prediabetes is characterized by defined skin manifestations.

## Material and methods

### Study design and population

We performed a single-center, epidemiological, cross-sectional, observational study. Data was collected during a single patient visit as part of the screening visit for the PREVIEW (Prevention of Diabetes through Lifestyle Intervention and Population Studies in Europe and around the World) (clinicaltrials.gov NCT01777893) Bulgarian cohort (12). The design, methods, patient characteristics, and primary outcomes have been discussed elsewhere (12–14). Recruitment was performed through community advertising and primary healthcare referral pathways targeting adults with overweight or obesity who were at increased metabolic risk. As a consequence, the screened population represents a high-risk cohort rather than a population-based sample, which explains the high proportion of individuals fulfilling criteria for prediabetes. The study participants were adult patients with obesity with or without concomitant prediabetes, all with skin Fitzpatrick phototype II and III. Patients were enrolled over six months. Eligible participants were adults aged 23–70 years with overweight or obesity (BMI ≥25 kg/m²), with or without prediabetes, as confirmed by an oral glucose tolerance test (OGTT) according to ADA criteria.

Primary outcome variables included the prevalence of skin diagnoses in both groups, with or without prediabetes, and secondary outcome variables included the grade of skin dryness, yellowish hue, and itch, as recorded on numeric scales, together with the skin tag count.

### Study procedures

All skin examinations were performed by a single trained dermatologist during a single study visit. Skin dryness, pruritus, and yellowish skin tone were assessed using visual numeric scales. Inter-rater reliability was not assessed, and these measures should be regarded as subjective clinical assessments.

Screening procedures included anthropometric measurements, resting blood pressure, and electrocardiography for those aged 55 years or older. A fasting venous blood sample was collected, followed by ingestion of a standard 75 g glucose solution (Oral Glucose Tolerance Test - OGTT), with plasma glucose assessed at baseline and after two hours to classify impaired fasting glucose or impaired glucose tolerance. Additional fasting samples were analyzed for safety parameters. Diabetes risk was further estimated using the Finnish Diabetes Risk Score.

### Statistical analysis

Data analysis was performed using SPSS v.20. A significance level of α=0.05 was applied, with results considered statistically significant at p<0.05. We used mean, median, standard deviation (SD), minimum, and maximum values for the descriptive statistics. Categorical variables are presented as absolute (n) and relative (%) values. The One-Sample Kolmogorov-Smirnov test was used to assess normality and evaluate the distribution of quantitative variables. The Chi-square test or Fisher’s Exact Test was used to determine relationships between categorical variables. The independent-samples t-test was used for normally distributed variables, while the Mann-Whitney test was used for non-normally distributed variables. Binary logistic regression was conducted to quantify factors associated with prediabetes, expressed as odds ratios (OR) indicating the strength and direction of associations. Binary logistic regression analyses were exploratory and unadjusted. No multivariable models controlling for BMI or biomechanical factors were performed. Therefore, the results should be interpreted with caution, acknowledging the potential for residual confounding.

### Ethical aspects

The study adhered to the ethical principles of the Declaration of Helsinki and the ICH-GCP (International Conference on Harmonization for Good Clinical Practice), to the extent possible and relevant. All subjects signed a written informed consent form before inclusion in the study. The PREVIEW study protocol and amendments were reviewed and approved by local Human Ethics Committees at all study sites. The collected information is handled in accordance with local regulations and the European Directive 95/46/CE (the directive on the protection of individuals with regard to the processing of personal data and on the free movement of such data). The trial was registered with ClinicalTrials.gov under NCT01777893.

## Results

### Study population characteristics

A total of 191 patients were included in the Bulgarian screening cohort of the PREVIEW study: 151 with obesity and prediabetes, and 40 patients with obesity without prediabetes. In the prediabetes group, there were 24 men (15.9%) with a mean age of 44.04 years (SD = 11.13, range: 27–67) and 127 women (84.1%) with a mean age of 46.47 years (SD = 12.22, range: 23–70). In the non-prediabetes group, there were five men (12.5%) with a mean age of 38.80 years (SD = 3.03, range: 35–43) and 35 women (87.5%) with a mean age of 47.31 years (SD = 11.19, range: 23–67), with no difference between groups regarding age and gender distribution.

A significant difference between patients with or without prediabetes was estimated for blood glucose levels at 0’ and at 120’ after OGTT, body weight, BMI, and diabetes risk (**Table 1A**).

**Table 1A:** Comparative analysis of anthropometric and laboratory parameters in subjects from both study groups.

Parameter	Group	N	Mean	Median	SD	p*
Blood glucose levels at 0’ (mmol/l)	Prediabetes	151	6,03	6,00	0,43	<0,001 (U)
	No prediabetes	40	4,94	5,00	0,41	
Blood glucose levels at 120’, (mmol/l)	Prediabetes	151	6,11	5,90	1,87	0,002 (t)
	No prediabetes	40	5,15	5,00	1,03	
Body weight, kg	Prediabetes	151	103,24	97,00	27,70	0,027 (U)
	No prediabetes	40	92,92	94,00	23,07	
Height, m	Prediabetes	151	1,66	1,65	0,09	0,909 (t)
	No prediabetes	40	1,66	1,65	0,08	
BMI, kg/m2	Prediabetes	151	37,30	35,70	8,31	0,001 (t)
	No prediabetes	40	32,05	32,09	10,19	
Diabetes Risk Score	Prediabetes	151	15,83	16,00	2,27	<0,001(U)
	No prediabetes	40	14,40	14,00	1,50	

BMI, Body Mass Index; N, number of subjects; SD, standard deviation.

*(t) - Independent Samples t-test; (U) - Mann-Whitney test

### Prevalence of skin conditions in patients with or without prediabetes

**Figure 1:**
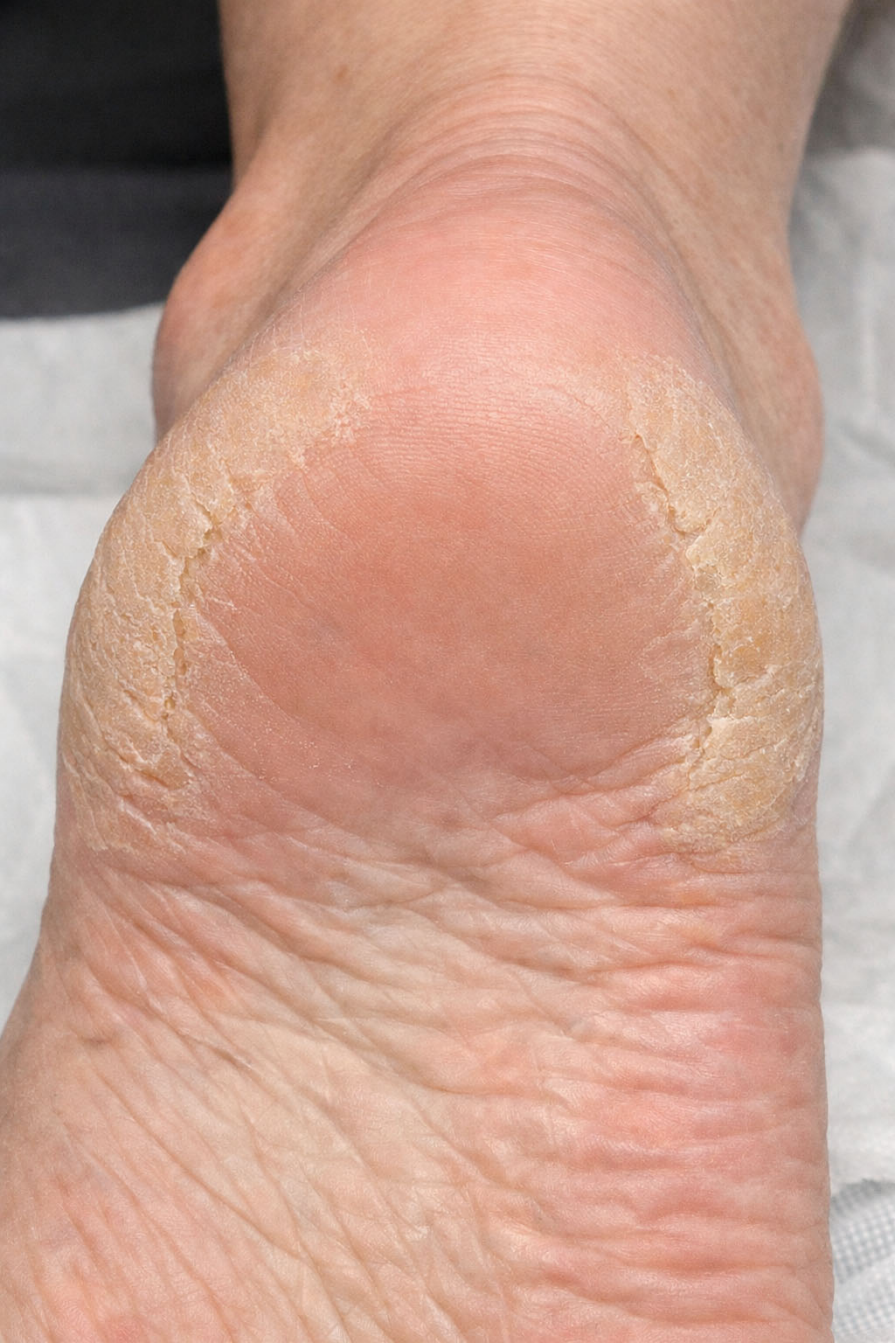
Conceptual illustration showing the typical shape and location of horseshoe-like plantar hyperkeratosis. HSLPH features a symmetrical, horseshoe-shaped area of thickened skin mainly on the outer plantar heel and the back edges, while the central calcaneal area remains relatively unaffected. This pattern distinguishes HSLPH from diffuse heel callus, which typically affects the central weight-bearing area. The lesion is often well-defined, long-lasting, and appears on both feet, indicating specific mechanical stress on altered plantar skin. The image is for illustration only and does not present primary clinical or experimental data. The image is intended for illustrative purposes only and does not represent primary experimental or clinical data. The authors reviewed and validated all elements for scientific accuracy.

A significant difference between groups was disclosed only for the horseshoe-like plantar hyperkeratosis (HSLPH) (**Figure 1**) (p=0.039), which was more prevalent in patients with prediabetes (**Table 1B**). HSLPH was defined clinically as a symmetrical, horseshoe-shaped hyperkeratotic thickening predominantly affecting the peripheral plantar heel with relative sparing of the central heel area, distinguishing it from diffuse or pressure-related callus.

**Table 1B:** Comparison of the prevalence of different skin conditions between patients with or without prediabetes.

Diagnosis	Group		Total	P
	Prediabetes n (%)	Without prediabetes n (%)		
Skin tags/acrochordons	74 (49,0)	17 (42,5)	91 (47,6)	0,464
Horseshoe-like plantar hyperkeratosis (HSLPH)	38 (25,2)	4 (10,0)	42 (22,0)	0,039
Onychomycosis	27 (17,9)	8 (20,0)	35 (18,3)	0,758
Varices, chronic venous insufficiency	29 (19,2)	4 (10,0)	33 (17,3)	0,171
Seborrheic dermatitis	26 (17,2)	4 (10,0)	30 (15,7)	0,265
Senile angiomas	23 (15,2)	4 (10,0)	27 (14,1)	0,398
Tinea pedis	22 (14,6)	2 (5,0)	24 (12,6)	0,104
Folliculitis	14 (9,3)	2 (5,0)	16 (8,4)	0,530
Rubeosis faciei	11 (7,3)	4 (10,0)	15 (7,9)	0,522
Acanthosis nigricans	11 (7,3)	1 (2,5)	12 (6,3)	0,466
Striae distensae	11 (7,3)	1 (2,5)	12 (6,3)	0,466
Intertrigo	10 (6,6)	2 (5,0)	12 (6,3)	1,000
Keratosis pilaris	6 (4,0)	3 (7,5)	9 (4,7)	0,399
Glossy skin over the pretibial area	6 (4,0)	3 (7,5)	9 (4,7)	0,399
Androgenic alopecia	8 (5,3)	0 (0,0)	8 (4,2)	0,208
Yellow nail discoloration	8 (5,3)	0 (0,0)	8 (4,2)	0,208
Hypertrichosis	5 (3,3)	0 (0,0)	5 (2,6)	0,586
Acne	5 (3,3)	0 (0,0)	5 (2,6)	0,586
Seborrheic keratoses	4 (2,6)	1 (2,5)	5 (2,6)	1,000
Palmar skin thickening	4 (2,6)	1 (2,5)	5 (2,6)	1,000
Tinea versicolor	3 (2,0)	1 (2,5)	4 (2,1)	1,000
Telogen effluvium (hair loss)	4 (2,6)	0 (0,0)	4 (2,1)	0,581
Erythrosis coli	3 (2,0)	0 (0,0)	3 (1,6)	1,000
Vitiligo	1 (0,7)	2 (5,0)	3 (1,6)	0,112
Histiocytomas	2 (1,3)	0 (0,0)	2 (1,0)	1,000

The most prevalent diagnoses in both groups were skin tags (soft fibromas, acrochordons), HSLPH, onychomycosis, varices/skin signs of chronic venous insufficiency, seborrheic dermatitis, senile angioma, and tinea pedis (mycotic foot infection).

Binary logistic regression disclosed that HSLPH was significantly associated with prediabetes(odds ratio (OR) of 3.03 (95% CI: 1.01–9.06, p = 0.048)), a threefold higher likelihood compared to participants without prediabetes. We then assessed the specificity, sensitivity, predictive value, and negative predictive value of HSLPH in distinguishing patients suffering from obesity with or without prediabetes (**Table 2**). Despite the reliable specificity of 90%, the sensitivity remained low (25%).

**Table 2:** Diagnostic test for evaluation of sensitivity, specificity, positive predictive value, and negative predictive value of the horseshoe-like plantar hyperkeratosis (HSLPH) as a screening test for prediabetes.

Statistic parameter	Value	95% CI
Sensitivity	25.17%	18.47% to 32.87%
Specificity	90.00%	76.34% to 97.21%
Positive Likelihood Ratio	2.52	0.95 to 6.64
Negative Likelihood Ratio	0.83	0.72 to 0.96
Disease prevalence	79.06%	72.59% to 84.60%
Positive Predictive Value	90.48%	78.27% to 96.16%
Negative Predictive Value	24.16%	21.71% to 26.79%

There was no statistical difference in skin tag quantity between groups (p=0.985). In addition, no significant correlation was found between skin tag count and body weight (Spearman’s rho R = 0.123, p=0.1), or BMI (Spearman’s rho R = 0.104, p=0.2). Skin dryness (p=0.464), itch (p=0.950), and yellowish skin hue (p=0.693) did not differ significantly between the two groups.

## Discussion

In this single-center cross-sectional pilot analysis of adults with obesity, horseshoe-like plantar hyperkeratosis (HSLPH) was the only cutaneous diagnosis that differed between participants with and without prediabetes. The prevalence of HSLPH was higher among those with prediabetes, and in logistic regression, it carried a roughly threefold higher odds of prediabetes (OR 3.03; 95% CI 1.01–9.06). The confidence interval was wide, and the lower bound was close to unity, so the estimate should be interpreted as a signal rather than a precise effect size.

Patients with prediabetes in our cohort were heavier. They had higher BMI, fasting glucose, and 2-hour glucose values, all of which increase mechanical load and alter plantar pressure distribution, i.e., well-known drivers of focal plantar hyperkeratosis (7, 8, 15). Microvascular and collagen glycation changes that begin early along the dysglycemia spectrum may further stiffen plantar skin and reduce its shock absorption, amplifying callus formation (16–18). Together, these pathways offer a reason why HSLPH is more prevalent in individuals with prediabetes and obesity.

From a diagnostic standpoint, HSLPH behaved as a specific but insensitive cue. Specificity approached 90%, whereas sensitivity was about 25%. The positive predictive value was high (~90%), but this largely reflects the high prevalence of prediabetes in this clinic-based sample of patients living with obesity (~79%). The negative likelihood ratio (~0.83) indicates that the absence of HSLPH does not substantially lower the probability of prediabetes. Despite high specificity, HSLPH had low sensitivity. Accordingly, HSLPH should not be considered a screening or diagnostic test. Instead, its presence may serve as a specific clinical “red flag” that prompts further metabolic evaluation in individuals suffering from obesity.

Importantly, recognition of HSLPH does not require specialist dermatological expertise. The characteristic distribution and morphology can be identified during routine foot inspection by non-dermatologists, including diabetologists, endocrinologists, podiatrists, primary care physicians, and other healthcare professionals. As foot examination is already standard practice in diabetes care, the presence of HSLPH may realistically influence case-finding behavior in metabolic clinics, podiatry services, and community or resource-limited healthcare settings where systematic laboratory screening for prediabetes is not always available.

Other common diagnoses, such as skin tags, onychomycosis, venous disease stigmata, seborrhoeic dermatitis, senile angiomas, and tinea pedis, were common in both groups and did not discriminate between prediabetes status. This pattern aligns with the literature, which links these conditions to obesity and aging per se rather than to dysglycemia alone (19–21). We could not find a between-group difference in the number of skin tags, and skin-tag counts did not correlate with weight or BMI. The absence of an association between skin tag count and BMI in the present study should be interpreted cautiously. All participants had overweight or obesity, resulting in a restricted metabolic and anthropometric range that may attenuate detectable differences once a high adiposity threshold is reached. In addition, clinical counting of skin tags at a single visit is susceptible to ceiling effects in individuals with numerous lesions, potentially further compressing variability and obscuring associations with continuous measures such as BMI. Several explanations are plausible. First, restricting the sample to individuals with obesity narrows the metabolic gradient; once a high adiposity threshold is crossed, additional weight gain may add little to the propensity for acrochordon formation (19, 22, 23). Second, counting skin tags at a single visit is prone to ceiling effects in patients with severe disease, thereby compressing variability (19, 23). Third, prior reports linking skin tags to insulin resistance often compare populations of patients living with obesity and healthy individuals (8, 22, 23); our within-obesity comparison is a stricter test and may therefore yield no differences.

The high prevalence of prediabetes observed in this cohort (79%) should be interpreted in light of both the targeted PREVIEW recruitment strategy and the substantial background burden of obesity and dysglycemia in Bulgaria. Available national and European data indicate a high prevalence of adult overweight and obesity, as well as a considerable proportion of undiagnosed impaired glucose regulation, particularly among middle-aged individuals. Accordingly, the present cohort reflects an enriched high-risk group rather than the general population.

From a mechanistic perspective, dysregulation of mTOR signaling provides a plausible biological link between obesity, prediabetes, and plantar hyperkeratosis. mTORC1 and mTORC2 integrate nutrient availability, insulin signaling, and mechanical stress responses (24–26). In states of insulin resistance and nutrient excess, sustained mTOR activation promotes keratinocyte proliferation, suppresses autophagy, and alters epidermal differentiation (26–28). Importantly, mTOR signaling intersects with mechanotransduction pathways responsive to increased plantar pressure, suggesting that metabolic and mechanical stress may converge to promote focal plantar hyperkeratosis in prediabetic individuals.

Subjective ratings of xerosis, pruritus, and yellowish skin tone also failed to separate groups. These features are ubiquitous in obesity and influenced by climate, bathing habits, emollient use, and observer expectation. A 10-point visual scale can misclassify subtle differences, and early glycation-related color shifts are difficult to score reliably in routine light without instrumental colorimetry (29, 30). The null findings here caution against overinterpreting these soft signs as indicators of prediabetes in a specific clinical setting.

In adults living with obesity, a persistent symmetric “horseshoe” pattern of plantar callusing may reasonably prompt consideration of metabolic testing, such as fasting glucose measurement or an oral glucose tolerance test. Conversely, reliance on skin tags, general xerosis, or a yellow hue to triage testing is unwarranted. Recognition of this pattern of plantar hyperkeratosis should therefore be considered part of general clinical awareness rather than a dermatology-specific screening strategy. Any clinician routinely inspecting the feet, namely diabetologists, podiatrists, primary care physicians, and other healthcare professionals, may encounter this sign and consider metabolic evaluation when appropriate.

This study has several limitations. The cross-sectional design precludes causal inference. Group sizes were imbalanced, limiting statistical power for less prevalent dermatoses. The logistic regression was unadjusted, and residual confounding by BMI, plantar pressure, footwear, and neuropathy cannot be excluded. Dermatological assessments were performed by a single observer without formal reproducibility testing.

## Conclusion

HSLPH represents a specific but insensitive cutaneous marker associated with prediabetes in adults with obesity. Its presence should prompt metabolic evaluation, but its absence cannot exclude dysglycemia. These findings should be regarded as hypothesis-generating and require validation in prospective studies that account for biomechanical and metabolic factors. These pilot results require future research that should be prospective and multimodal: standardized plantar pressure mapping, photographic grading of callus distribution, objective color measurements, and explicit adjustments for BMI, footwear, and neuropathy. Interventional studies would be most valuable, such as testing whether weight loss, pressure-redistributing insoles, or footwear modifications reduce HSLPH and whether such changes correlate with improvements in glycemic indices.

## Data Availability

The original contributions presented in the study are included in the article/supplementary material. Further inquiries can be directed to the corresponding author.
